# Letter regarding “American Venous Forum Clinical Practice Guideline on the care of patients with upper extremity deep venous thrombosis”

**DOI:** 10.1016/j.jvsv.2026.102513

**Published:** 2026-05-06

**Authors:** Tjep Hoedemakers, Lara.T. Mascini, Bart-Jeroen Petri, Gert J. de Borst, Eline S. van Hattum

**Affiliations:** Department of Vascular Surgery, University Medical Center Utrecht, Utrecht, The Netherlands

We commend the American Venous Forum for its comprehensive clinical practice guidelines regarding the care of patients with upper extremity deep venous thrombosis (UEDVT).^1^ Although we agree with the majority of the recommendations, we respectfully question Guidelines 4.4 and 4.5, which recommend magnetic resonance venography (MRV) as the preferred advanced imaging modality over computed tomography venography (CTV) following an inconclusive duplex ultrasound.

The committee’s recommendation appears to be based on a single study,^2^ in which not MRV, but highly specialized magnetic resonance (MR) noncontrast thrombus imaging modalities were assessed on their ability to diagnose UEDVT, using CTV as the reference standard. In addition to the more obvious practical limitations of MR imaging such as availability, cost, and acquisition time, over 10% of patients were excluded due to interfering MR artifacts. Notably, the two false-negative MR cases in the study were correctly diagnosed with CTV, underscoring its sensitivity.

In appraising MRV, disadvantages such as its diagnostic limitations were disregarded. Contrary to the committee’s recommendation, a review by Kraaijpoel et al concluded that MRV is expensive, time-consuming, and lacks sufficient evidence to support its routine use for the diagnosis of UEDVT.^3^

The committee’s recommendation to use CTV only when MRV is unavailable was based on presumed lack of current evidence regarding CTV. Although larger studies on diagnostic accuracy are lacking, the American College of Radiology considers CTV appropriate for the assessment of UEDVT.^4^

Practical advantages of CTV include its widespread availability, time-efficiency, and relatively low cost. Furthermore, CTV allows simultaneous assessment of potential causes of UEDVT. Venous thoracic outlet syndrome is the most common cause of unprovoked UEDVT, for which CTV allows excellent assessment.^5^ In a previous study, CTV demonstrated superior reliability compared with MRV with regard to assessment of venous compression in the thoracic outlet.^6^

Therefore, in the absence of comparative studies between CTV and MRV, we propose recommending CTV rather than MRV as the preferred subsequent diagnostic modality after an inconclusive duplex ultrasound in patients who are clinically suspect for UEDVT. We are interested in the committee’s perspective.

## Declaration of generative AI and AI-assisted technologies in the writing process

During the preparation of this work the authors used OpenAI-ChatGPT in order to rephrase some sentences. After using this tool/service, the authors reviewed and edited the content as needed and take full responsibility for the content of the publication.

## Funding

None.

## Disclosures

None.
